# Perioperative anesthesia management for a child with congenital adrenal cortical hyperplasia undergoing thoracoscopic repair of atrial septal defect: a case report and literature review

**DOI:** 10.1186/s12871-026-04071-9

**Published:** 2026-07-03

**Authors:** Jiao Wang, Shiqian He, Xiaoyi Pang, Weijie Zhang

**Affiliations:** Department of Anesthesiology, Mianyang 404 Hospital, Mianyang, Sichuan 621000 China

**Keywords:** Congenital adrenal cortical hyperplasia, Atrial septal defect, Thoracoscopic surgery, Pediatric anesthesia, Perioperative anesthesia management

## Abstract

**Background:**

Congenital adrenal cortical hyperplasia (CAH) includes a group of autosomal recessive genetic disorders characterized by impaired synthesis of adrenal corticosteroid hormones. The clinical manifestations associated with CAH commonly include electrolyte imbalance, refractory hypotension, metabolic acidosis, hypoglycemia, and adrenal crisis. The coexistence of endocrine insufficiency and cardiopulmonary abnormalities complicates clinical management in children with CAH undergoing thoracoscopic atrial septal defect (ASD) repair for congenital heart disease. Although thoracoscopic procedures have advantages because of their minimally invasive nature, they also involve risks such as pneumothorax, low-tidal-volume ventilation, and postural changes, which may aggravate hemodynamic fluctuations. These factors considerably increase the difficulty of perioperative management and the risk of complications. Therefore, precise and individualized perioperative anesthesia management is essential. To maintain hemodynamic and respiratory function and ensure surgical safety, comprehensive preoperative assessment, standardized glucocorticoid stress replacement, optimized anesthesia strategies, advanced multi-dimensional monitoring, and careful maintenance of circulatory and internal environmental stability are required.

**Case presentation:**

This article describes a clinical case of a 21-month-old infant diagnosed with CAH complicated by ASD. After assessment by a multidisciplinary team (MDT), an individualized perioperative anesthesia strategy was developed and applied. Thoracoscopic ASD repair was successfully performed through comprehensive management, including preoperative correction of electrolyte imbalance, intraoperative hormone replacement therapy, continuous monitoring of the internal milieu and hemodynamic stability, and postoperative optimization of pain control and anti-infective therapy. No significant complications occurred during the perioperative period.

**Conclusion:**

Effective perioperative management should address insufficient and excessive secretion of adrenal cortical hormones, as well as the effects of long-term exogenous hormone therapy in children with CAH undergoing cardiac surgery. In addition, maintenance of endocrine homeostasis and hemodynamic stability is critical for reducing the risk of adverse events. This case further suggests that the clinical value of management lies in integrating endocrine preparation with cardiac anesthesia and postoperative surveillance, rather than in any single isolated intervention.

## Background

Congenital adrenal cortical hyperplasia (CAH) is a group of autosomal recessive genetic disorders caused by deficiencies in key enzymes involved in adrenal corticosteroid synthesis [1]. These deficiencies impair the production of glucocorticoids and mineralocorticoids, leading to excessive androgen secretion [2]. Consequently, affected patients may develop several critical clinical manifestations, including electrolyte imbalance, refractory hypotension, metabolic acidosis, hypoglycemia, and potentially life-threatening adrenal crisis [3]. The perioperative period carries a high risk of acute adrenal crisis, which may become a severe and life-threatening complication. Atrial septal defect (ASD) is one of the most common congenital heart defects in children [4]. Thoracoscopic repair of ASD is associated with less surgical trauma and favorable early postoperative recovery [5]. However, intraoperative factors such as artificial pneumothorax, low-tidal-volume ventilation, and lateral decubitus positioning may substantially affect hemodynamic stability and systemic circulation.

A complex clinical scenario is formed when children with CAH are complicated by ASD, involving both endocrine and metabolic abnormalities and structural heart defects. Anesthesia management for these patients must simultaneously consider adrenal insufficiency, cardiac shunting, hemodynamic instability, and metabolic disturbances. This complexity results in a markedly higher risk compared with patients with a single pathology. At present, literature on anesthesia protocols for children with CAH undergoing thoracoscopic ASD repair remains limited, resulting in a lack of standardized management approaches. Based on a clinical case and literature review, this article aims to summarize perioperative anesthesia strategies for such cases and provide practical reference for clinical practice. The educational value of this case lies in the need to manage CAH-related adrenal insufficiency simultaneously with thoracoscopic cardiac surgery and cardiopulmonary bypass, requiring coordinated steroid replacement, invasive monitoring, ventilation adjustment around bypass, and postoperative endocrine and respiratory surveillance.

The key educational message is that apparently routine components of care, including steroid supplementation, cardiac anesthesia, cardiopulmonary bypass management, ventilation interruption and resumption, and postoperative extubation planning, require closer coordination when CAH is present. In this context, the case provides a practical framework for anticipating endocrine decompensation during cardiac surgery rather than merely describing standard anesthetic care.

## Case presentation

A 21-month-old female infant, weighing 9 kg, was admitted to the hospital because of a history of congenital heart disease identified more than one year earlier and ASD diagnosed more than one week before admission. The child was diagnosed with CAH at 3 months of age and had received long-term oral replacement therapy consisting of hydrocortisone (1.25 mg per dose, administered three times daily) and fludrocortisone (0.05 mg per dose, twice daily). Admission diagnosis: Congenital heart disease: ASD (secundum type); and CAH. Physical examination showed a body temperature of 36.4 °C, pulse of 128 beats per minute, respiratory rate of 26 breaths per minute, and blood pressure of 95/60 mmHg. No cyanosis was observed; the thorax was symmetrical; bilateral lung breath sounds were clear; heart rhythm was regular; a grade 2–3/6 systolic murmur was heard at the 2nd to 3rd intercostal spaces along the left sternal border; and vulvar hermaphroditism was noted. Laboratory tests: Routine blood tests, electrolytes, blood glucose, liver and kidney function, and coagulation function were all within normal ranges. Endocrine examination: Cortisol level was 648 nmol/L, aldosterone was 36.8 pg/mL, adrenocorticotropic hormone was 76.5 pg/mL, thyroid-stimulating hormone was 6.27 uIU/mL, and free T3 was 7.38 pmol/L. Cardiac color Doppler ultrasound findings suggested congenital heart disease, ASD (secundum central type), defect size of 9 mm, left-to-right shunt at the atrial level, right heart enlargement, mild pulmonary hypertension, increased velocity across the pulmonary valve, mild tricuspid regurgitation, small pericardial effusion, and left ventricular ejection fraction of 71%.

Preoperatively, a multidisciplinary team (MDT), including anesthesiology, cardiac surgery, endocrinology, pediatrics, the pediatric intensive care unit (PICU), and the clinical laboratory, conducted comprehensive preoperative assessment and preparation for the child. Three days before surgery, the oral hydrocortisone dose was gradually increased to 5 mg three times daily. In addition, on the day before surgery, intravenous hydrocortisone was administered at 1 mg/kg every 12 h.

After the pediatric patient entered the operating room, all standard monitors were connected. These included electrocardiography (ECG), non-invasive blood pressure (NIBP), pulse oxygen saturation (SpO_2_), and temperature monitoring. Anesthesia induction was performed using midazolam (0.1 mg/kg), sufentanil (1 µg/kg), propofol (2 mg/kg), and rocuronium (0.6 mg/kg). After loss of consciousness, mask ventilation was performed for 3 min, followed by intubation with a 4.0-size tracheal tube inserted to a depth of 12 cm for mechanical ventilation. The ventilation parameters were set as follows: tidal volume 10 mL/kg, respiratory rate of 25 breaths per minute, inspiratory-expiratory ratio of 1:2, and fraction of inspired oxygen (FiO_2_) of 50%. The partial pressure of end-tidal carbon dioxide (PETCO_2_) was maintained between 35 and 45 mmHg. Anesthesia was maintained through continuous intravenous infusion of propofol (8–10 mg·kg^–1^·h^–1^) and dexmedetomidine (0.4 µg·kg^–1^·h^–1^), supplemented by intermittent intravenous boluses of rocuronium (0.1 mg/kg) and sufentanil (0.5 µg/kg) as needed. Meanwhile, hydrocortisone was administered by continuous infusion at 3.2 mg/h. Intraoperative invasive arterial blood pressure (IABP) was continuously monitored through radial artery cannulation to evaluate hemodynamic fluctuations in real time. Serial blood gas analyses were performed to dynamically assess the internal environment and maintain electrolyte and acid-base homeostasis. Central venous pressure (CVP) was monitored through internal jugular vein catheterization, with the target maintained at 8–12 cmH_2_O.

Intraoperatively, the patient’s vital signs remained stable, indicating well-maintained internal homeostasis. Reexamination of intraoperative laboratory results showed a serum cortisol level of 784 nmol/L, aldosterone of 43.2 pg/mL, and adrenocorticotropic hormone of 13.1 pg/mL.

During thoracoscopic ASD repair, mechanical ventilation was carefully adjusted during the non-cardiopulmonary bypass periods, with settings including tidal volume of 6–8 mL/kg, respiratory rate of 25–30 breaths/min, and inspiratory-expiratory ratio of 1:1.5. The fraction of FiO2 was adjusted between 60% and 70% to maintain SpO2 and PETCO2 within normal physiological limits. Mechanical ventilation was performed for 144 min from induction of anesthesia to the initiation of cardiopulmonary bypass, suspended during cardiopulmonary bypass, and resumed for 107 min from the termination of cardiopulmonary bypass to the end of surgery. Postoperatively, mechanical ventilation was continued for an additional 120 min before extubation. Throughout the operation, no obvious episodes of hemodynamic instability or hypoxemia occurred.

After definitive ASD repair, intraoperative transesophageal echocardiography (TEE) confirmed complete closure of the defect. To correct atelectasis, pulmonary re-expansion was performed, and ventilator parameters were returned to conventional bilateral ventilation mode. Cardiopulmonary bypass was used during the procedure, with a total bypass duration of 53 min. After cardiac resuscitation, dobutamine was continuously infused at 3 µg/kg/min. No intraoperative blood transfusion was required.

After the procedure, the child was transferred to the PICU, and endotracheal intubation was maintained to provide continued sedation, analgesia, and mechanical ventilation. Continuous postoperative monitoring included IABP, heart rate, urine output, blood gas parameters, electrolytes, and blood glucose levels. Pain was effectively managed using patient-controlled intravenous analgesia (PCIA). On postoperative day 1, hydrocortisone was administered intravenously at a dose of 2 mg/kg every 6 h, with gradual dose tapering beginning on postoperative day 2 until the preoperative oral dose was reached. The endotracheal tube was successfully removed 120 min after surgery. The patient remained in the ICU for 5 days and was then transferred to the general ward, without any episodes of adrenal crisis, heart failure, pulmonary complications, or electrolyte disturbance. The total hospital length of stay was 17 days. Follow-up at one month confirmed complete clinical recovery.

## Discussion

CAH, an autosomal recessive genetic disorder, is characterized by deficiencies in adrenal steroidogenic enzymes. Among its different subtypes, 21-hydroxylase deficiency is the most common, accounting for more than 90% of clinical cases. These enzymatic defects impair cortisol synthesis and reduce negative feedback inhibition, thereby stimulating compensatory hypersecretion of hypothalamic corticotropin-releasing hormone and pituitary adrenocorticotropic hormone [6]. The perioperative period presents a substantial risk for these patients, making them vulnerable to severe complications such as electrolyte disturbance, refractory hypotension, and acute adrenal crisis [2]. Thoracoscopic ASD repair has been widely accepted for pediatric congenital heart disease because of its minimally invasive nature and rapid postoperative recovery. However, this procedure requires high surgical precision and markedly affects respiratory and hemodynamic homeostasis. In addition, establishment of intraoperative artificial pneumothorax further increases the complexity of anesthetic management [5, 7]. Based on the present case and relevant literature, the essential points of anesthetic management for children with CAH complicated by ASD and undergoing thoracoscopic cardiac surgery are summarized as follows. Compared with routine thoracoscopic ASD anesthesia, the main clinical value of this case lies in integrating stress-dose glucocorticoid replacement with cardiac surgical management requiring cardiopulmonary bypass in an infant with CAH. This case emphasizes that anesthesiologists should anticipate adrenal crisis, electrolyte and glucose instability, and exaggerated hemodynamic responses, particularly during induction, separation from bypass, and early postoperative recovery.

Although stress-dose glucocorticoid administration itself is not novel, its use during thoracoscopic ASD repair with cardiopulmonary bypass is clinically meaningful because bypass, ventilation suspension, rewarming, cardiac resuscitation, and early postoperative recovery may all obscure or mimic the signs of adrenal crisis. Early recognition may therefore be difficult if the team relies only on routine cardiac anesthesia indicators. The practical implication is that blood pressure, glucose, potassium, acid-base status, cortisol/ACTH changes, and response to inotropic support should be interpreted together in children with CAH.

In previously published literature, CAH has mainly been discussed from the perspective of endocrine diagnosis, chronic hormone replacement, and prevention or treatment of adrenal insufficiency [1–3, 6, 10, 11]. In contrast, reports on thoracoscopic congenital cardiac surgery mainly focus on minimally invasive cardiac techniques, one-lung or low-tidal-volume ventilation, artificial pneumothorax, and hemodynamic effects related to cardiopulmonary bypass [5, 7–9]. These two areas are generally addressed separately. The present case differs from these reports because the anesthetic team had to manage endocrine insufficiency and cardiopulmonary surgical stress concurrently in an infant. Therefore, its additional value is that it connects these two management issues and shows how steroid replacement, monitoring, ventilation, vasoactive support, and postoperative surveillance can be integrated into one perioperative plan.

### Preoperative assessment and preparation

For children with CAH and concurrent ASD undergoing thoracoscopic ASD repair, adequate preoperative assessment and standardized preparation are prerequisites for ensuring perioperative anesthetic safety. The evaluation should include: assessment of adherence to hormone replacement therapy and confirmation that hormone levels are adequate to tolerate surgical stress; assessment of ASD size, shunt volume, and cardiopulmonary function; evaluation of the effects of intraoperative thoracoscopic pneumothorax, low-tidal-volume ventilation, and postural changes on hemodynamics; monitoring of the internal milieu of the children, particularly sodium (Na^+^), potassium (K^+^), blood glucose, and pH levels; and examination of airway conditions, nutritional status, and other relevant indicators [3, 8].

An MDT including anesthesiology, cardiac surgery, endocrinology, pediatrics, PICU, and the clinical laboratory was established. This MDT collaboration enabled clear definition of the perioperative hormone stress replacement protocol, as well as the identification and emergency response protocols for adrenal crisis. The internal environment of the children was optimized, and water-electrolyte and acid-base disorders were corrected. Active measures were taken to control pulmonary infections and adjust cardiac function. In addition, blood products, vasoactive drugs, and perioperative monitoring equipment were prepared, providing a solid foundation for the smooth progression of anesthesia and surgery. In this case, CAH directly influenced perioperative decision-making: the steroid regimen was intensified before surgery, hydrocortisone was continued intravenously during anesthesia, and serial blood gas, glucose, electrolyte, cortisol, and ACTH assessments were used to guide timely adjustment, rather than relying only on routine cardiac anesthesia monitoring.

The MDT discussion was therefore not merely a routine preoperative consultation. It was used to define the timing and route of hydrocortisone administration, determine the indicators of suspected adrenal crisis, prepare rescue hydrocortisone and vasoactive medications, and coordinate postoperative transfer to the PICU. This preparation was important because hypotension after induction or after separation from bypass could result from myocardial dysfunction, residual shunt, hypovolemia, pulmonary hypertension, or adrenal insufficiency, and these possibilities require different immediate responses.

### Principles of anesthesia induction and maintenance

Preoperative supplementation of glucocorticoids according to standard protocols is necessary, together with correction of electrolyte imbalance and metabolic acidosis in children with CAH complicated by ASD undergoing thoracoscopic ASD repair, to ensure that the body is in a favorable condition during anesthesia induction. A stable and gentle induction technique should be used to reduce intubation-related stress, avoid myocardial depression, and prevent fluctuations in pulmonary vascular resistance, thereby maintaining the stability of intracardiac shunting. During anesthesia maintenance, continuous hormone replacement throughout the procedure is critical. This should be accompanied by accurate regulation of anesthetic depth, rational use of ventilation strategies, and strict control of airway pressure and intrathoracic pressure to prevent hypercapnia and hypoxemia. In addition, close monitoring of circulatory function, blood gas parameters, blood glucose levels, and changes in the internal milieu is essential. Anesthetic agents with minimal cardiovascular effects should be selected to prevent increased right heart load and hemodynamic disorders [9]. Moreover, drugs that inhibit adrenal cortical function, such as etomidate, should be strictly avoided to prevent worsening of hormone deficiency and increased risk of adrenal crisis [10, 11].

In this case, anesthetic agents including midazolam, sufentanil, rocuronium, propofol, and dexmedetomidine were selected for induction and maintenance of anesthesia. These agents have rapid onset and mild effects on the cardiovascular system, contributing to stable vital signs throughout the procedure and the absence of related complications. Invasive arterial pressure monitoring, CVP monitoring, repeated blood gas analysis, and TEE were used to rapidly identify hypotension, acid-base disturbance, electrolyte abnormalities, or residual shunting during thoracoscopy and cardiopulmonary bypass.

The anesthetic technique was selected to minimize abrupt sympathetic stimulation and myocardial depression. For this reason, induction was performed gently, anesthetic depth was maintained with titratable intravenous agents, and etomidate was avoided because even transient adrenal suppression could be unfavorable in a child with impaired endogenous cortisol synthesis. Monitoring was also expanded beyond routine vital signs: repeated blood gas analysis was used to detect metabolic acidosis or hypoglycemia early, electrolyte testing was used to identify hyperkalemia or hyponatremia, and TEE was used to distinguish cardiac causes of hemodynamic instability from endocrine or metabolic causes.

### Perioperative hormone replacement and stress management

In children with CAH, glucocorticoid replacement therapy is the cornerstone of perioperative management. During surgical stress, children with CAH have insufficient endogenous cortisol synthesis and therefore require exogenous glucocorticoid supplementation to meet increased stress demands and effectively prevent adrenal crisis. Relevant studies have indicated that, because of individual differences in drug metabolism and polymorphisms of glucocorticoid receptor genes, the glucocorticoid dose required for children under stressful conditions, such as surgery and trauma, can reach two to three times the physiological dose per unit body weight [12]. The daily dose of hydrocortisone can be increased by 3 to 6 times to prevent adrenal crisis induced by surgical or traumatic stress [13]. The dosage of glucocorticoid replacement should be adjusted according to the intensity of surgical stress during the perioperative period in children with CAH. Hydrocortisone should be supplemented for moderate-intensity surgeries (e.g., ASD repair), in stages before, during, and after the operation to ensure adequate hormone levels under stressful conditions [14, 15]. Hormone replacement dosage must be individualized to avoid adrenal crisis due to insufficient dosage or metabolic abnormalities caused by excessive dosage. In addition, close monitoring of adrenocorticotropic hormone and cortisol levels is essential during the perioperative period. The hormone dosage should be adjusted promptly according to the child’s vital signs and changes in electrolytes and blood glucose, to ensure that replacement therapy meets clinical requirements.

The oral hydrocortisone dose was gradually increased three days before surgery. The administration route was changed to intravenous injection on the day before surgery, at a dose five times the usual dose. Hydrocortisone was continuously infused intravenously during surgery, and the dose was gradually tapered to the preoperative oral dose after the operation. No adrenal crisis occurred in the child throughout the perioperative period. The practical lesson is that steroid supplementation should be planned before surgical stress occurs, maintained during bypass and postoperative recovery, and tapered only after hemodynamic and metabolic stability has been confirmed.

The staged regimen used in this case reflected the expected stress trajectory of cardiac surgery. Increasing the oral dose before surgery was intended to improve preoperative reserve; switching to intravenous hydrocortisone reduced the risk of unreliable gastrointestinal absorption around anesthesia; continuous intraoperative infusion avoided gaps during thoracoscopy and cardiopulmonary bypass; and postoperative tapering was delayed until extubation and metabolic stability were achieved. This rationale explains why the hormone plan was individualized rather than simply continuing the home replacement dose.

### Prevention and control of complications

Multi-dimensional intensive monitoring during surgery is essential when children with CAH undergo thoracoscopic ASD repair, to prevent complications such as arrhythmia, electrolyte imbalance, acidosis, hypotension, hypoxia, hypoglycemia, and adrenal crisis. This monitoring includes ECG, IABP, CVP, SpO_2_, PETCO_2_, electrolytes, blood glucose levels, blood gas analysis, hormone levels, and TEE, allowing real-time evaluation of the respiratory and circulatory status of the children as well as changes in their internal environment. In addition, continuous monitoring of hormone levels, electrolytes, and cardiac function remains necessary during the postoperative recovery period in children with CAH. Nutritional support and respiratory tract management should also be strengthened to facilitate recovery. Acute adrenal crisis is the most severe complication during the perioperative period of CAH. High vigilance should be maintained for adrenal crisis when children present with unexplained hypotension, bradycardia, hypoglycemia, hyperkalemia, fever, or poor mental response [16]. Once diagnosed, a high dose of hydrocortisone (5–10 mg/kg) should be administered intravenously immediately, together with prompt correction of hypotension, electrolyte imbalance, and acidosis [17].

In this case, the child did not develop any serious complications during the perioperative period and achieved smooth recovery through comprehensive preoperative preparation, strict intraoperative monitoring, and standardized perioperative hormone replacement therapy. The hemodynamic course remained stable after separation from cardiopulmonary bypass with low-dose dobutamine support, and no further vasoactive escalation was required.

For anesthesiologists, the main lesson from this case is that unexplained hypotension, acidosis, electrolyte disturbance, or delayed recovery after thoracoscopic cardiac surgery in a child with CAH should not be immediately attributed to cardiac or respiratory causes alone. Adrenal crisis should remain in the differential diagnosis throughout induction, separation from bypass, and early PICU care. Early multidisciplinary communication and predefined rescue plans may shorten the time to appropriate treatment if instability occurs.

### Strengths and limitations

Anesthetic management for thoracoscopic ASD repair in a child with CAH was successfully achieved through thorough preoperative evaluation and preparation, individualized anesthetic planning, standardized hormone replacement therapy, and rigorous perioperative monitoring. No serious complications, such as adrenal crisis or arrhythmias, occurred, and the child had a smooth postoperative recovery, providing useful clinical experience. The key points of anesthetic management are summarized as follows: (1) Preoperative MDT collaboration comprehensively assessed the effectiveness of CAH hormone replacement and cardiac function, with a focus on correcting electrolyte and hormone imbalance to establish a solid foundation for anesthesia and surgery. (2) Anesthesia induction and maintenance should use drugs with minimal cardiovascular effects and no interference with adrenal function, while dosage and administration rates should be strictly controlled to prevent significant hemodynamic fluctuations. (3) Perioperative glucocorticoid replacement dosage should be individualized according to the intensity of surgical stress, with enhanced multi-dimensional monitoring and timely intervention for prevention and management of complications. (4) Postoperatively, continuous hormone management, respiratory tract management, and nutritional support are essential to ensure smooth recovery. Given the limited literature on CAH combined with thoracoscopic congenital cardiac surgery in infants, this report provides a practical perioperative workflow rather than evidence for a universal protocol.

Thus, the educational contribution of this report is a case-based checklist: confirm the adequacy of preoperative hormone replacement, intensify glucocorticoid coverage before major surgical stress, avoid adrenal-suppressive anesthetic drugs, monitor glucose/electrolytes/acid-base status together with cardiac parameters, document ventilation and bypass periods clearly, and continue postoperative endocrine surveillance until the child is clinically stable.

The limitations were as follows: (1) The generalizability of the conclusions requires verification by more clinical cases, because this is a single-center, single-case report with a small sample size. (2) The optimal dosage and administration method could not be established, because there was no comparative analysis of different hormone replacement regimens. (3) The postoperative follow-up period was limited, lacking long-term observation of hormone levels and recovery of cardiac function in the child. (4) Comprehensive external hospital medical records and CAH subtype confirmation by genetic testing were unavailable. Future multi-center, large-sample clinical studies are needed to explore optimal perioperative anesthetic management strategies for children with CAH undergoing cardiac surgery, optimize hormone replacement regimens, and improve the safety and effectiveness of anesthesia.

## Conclusion

Children with CAH are at high risk during thoracoscopic ASD repair, and their perioperative anesthesia management is relatively complex. This single case suggests that careful MDT-based preoperative optimization, planned stress-dose glucocorticoid replacement, invasive hemodynamic and metabolic monitoring, ventilation management around cardiopulmonary bypass, and structured postoperative surveillance may help reduce perioperative risk. Comprehensive preoperative assessment, standardized glucocorticoid stress replacement therapy, precise regulation of hemodynamics, continuous and strict monitoring of internal environmental homeostasis, and effective supplementary postoperative analgesia management are the core measures to ensure perioperative safety in these children. However, because this is a single-case report, these observations should be interpreted as practical clinical experience rather than generalizable evidence, and further cases are required to refine perioperative protocols for this rare clinical scenario.

## Data Availability

All data generated or analysed during this study are included in this published article. The raw clinical records of the patient are not publicly available to protect patient privacy and confidentiality.

## Abbreviations

CAH: Congenital adrenal cortical hyperplasia
ASD: Atrial septal defect
MDT: Multidisciplinary team
PICU: Pediatric intensive care unit
ECG: Electrocardiography
NIBP: Non-invasive blood pressure
SpO_2_: Pulse oxygen saturation
FiO_2_: Fraction of inspired oxygen
PETCO_2_: Partial pressure of end-tidal carbon dioxide
IABP: Invasive arterial blood pressure
CVP: Central venous pressure
TEE: Transesophageal echocardiography
PCIA: Patient-controlled intravenous analgesia
CPB: Cardiopulmonary bypass
